# Traumatic spinal cord injury caused by low falls and high falls: a comparative study

**DOI:** 10.1186/s13018-021-02379-5

**Published:** 2021-03-27

**Authors:** Zhen-Rong Zhang, Yao Wu, Fang-Yong Wang, Wen-Jing Wang

**Affiliations:** 1 School of Rehabilitation, Capital Medical University, No. 10, Jiaomen North Road, Fengtai District, Beijing, 100068 People’s Republic of China; 2 Department of Spine Surgery, Beijing Bo’ai Hospital, China Rehabilitation Research Center, Beijing, People’s Republic of China; 3 Department of Occupational Therapy, Beijing Bo’ai Hospital, China Rehabilitation Research Center, Beijing, People’s Republic of China

**Keywords:** Spinal cord injury, Fall, Epidemiology, Functional outcome, Older people, Workplace, Length of stay

## Abstract

**Background:**

Quite a few traumatic spinal cord injuries (TSCI) were caused by falls. However, the comparison of different causes of TSCI or the epidemiological characteristics of TSCI caused by falls of different heights are rare. This study investigated the epidemiological characteristics of TSCI caused by falls and conducted a comparison between low falls and high falls.

**Method:**

Data from cases with TSCI admitted to China Rehabilitation Research Center from 2010 to 2019 were collected, including age, gender, occupation, cause, neurological level, and severity of the injury in admission, combined injuries, complications, and rehabilitation length of stay. Mann-Whitney *U* and chi-square (*χ*^2^) tests were used to assess the differences between two groups at a statistical significance level of 0.05.

**Result:**

A total of 1858 TSCI cases were included and 41.7% were caused by falls, 11.4% by low falls and 30.3% by high falls, respectively. Patients with fall-induced TSCI were older and had a shorter rehabilitation length of stay than those with non-fall-induced TSCI. Patients with high fall-induced TSCI were younger and more likely to suffer from paraplegia, severer injuries, and combined injuries, and had longer time from injury to rehabilitation and rehabilitation length of stay, compared with patients with low fall-induced TSCI.

**Conclusion:**

Falls is the leading causes of TSCI and high fall is becoming more common. Attention not only should be paid to high falls for the severe injury and longer hospitalization, but also low falls due to the higher neurological level of the injury and the aging of population in China.

## Background

A fall is defined as an injury to a person that occurs after landing due to slipping, tripping, stumbling, collision, or after descent from a higher place, such as a furniture, ladder, scaffold, building, work area, or other elevated place [1]. Falls are a common and serious problem, which causes an enormous burden to both the family and society in terms of healthcare and costs [2]. It has been reported that the incidence of fall-related injuries among older Chinese people ranged from 0.6 to 19.5% and the cost ranged from US$16 to US$3812 per person per fall [3, 4]. In particular, falls from height, which are usually unintentional (92.2%), result in higher disability and mortality rates [5].

Falls play an important role in traumatic spinal cord injury (TSCI). The percentage of TSCI cases associated with falls increased from 28% in 1997–2000 to 66% in 2010–2012 in patients aged 65 years or older [6]. Injuries caused by falls represented 49.8% of cases in Saint Petersburg from 2012 to 2016, and elderly women more often suffered from low falls than men [7]. A systematic review on an epidemiological survey of spinal cord injury (SCI) in China showed that falls resulted in 23.9–65.3% of all SCI cases, and that falls were the primary causation of injury in most cities of China [8]. Moreover, another retrospective investigation in Northwest China from 2014 to 2018 showed that falls resulted in 85.1% (low falls 47.7%, high falls 37.3%) of all SCI cases [9]. Although epidemiological features of TSCI vary dramatically around the world, it can still be inferred that falls have a great influence on the occurrence of TSCI. In 2013, the World Health Organization published *International Perspectives on Spinal Cord Injury*, which defines falling above 1 m was classified as high fall, and below 1 m was classified as low fall [10]. This definition may distinguish low falls and high falls from the causes and characteristics of the injury.

A specific survey of the causes of falls resulting in SCI revealed that different demographic characteristics were present in patients and that varied situations led to the falls [11]. Here, we conduct a retrospective study and provide an in-depth description of the epidemiological characteristics of TSCI caused by falls in order to understand the epidemiology of TSCI resulting from both falls and non-falls, and to examine demographic and clinical characteristics of patients with TSCI caused by low falls and high falls. We believe this comparative study could provide evidence for preventing or reducing fall-induced TSCI in China and helping clinicians diagnose and treat patients caused by falls professionally.

## Methods

### Aim

This study investigated the epidemiological characteristics of TSCI caused by falls and non-falls and conducted a comparison between low falls and high falls.

### Participants and processes

Detailed information of patients with TSCI was obtained from the Medical Record System of China Rehabilitation Research Center, Beijing, China from 1 January 2010 to 31 December 2019, including age, gender, occupation, cause, neurological level and severity of the injury in admission, vertebra fracture and dislocations, complications, and rehabilitation length of stay (LOS). Two independent researchers examined the medical records and imaging examination to identify cases with TSCI caused by falls. Falling below 1 m was classified as low fall, and above 1 m was classified as high fall. Any disagreement was resolved after discussing with each other and reached an agreement. After grouping patient, cases resulted from falls were analyzed in depth.

### Measures and definition

The participants were divided into five age groups: 0–14, 15–29, 30–44, 45–59, and ≥ 60 years. The etiologies included falls and non-falls (including motor vehicle collisions, object striking, sports-related, assault, work-related, and others). Neurological functions were evaluated according to the American Spinal Injury Association Impairment Scale [12] as AIS grade A, B, C, D, and E (AIS grade A for complete SCI, AIS grade B, C, D, and E for incomplete SCI) [13].

### Statistical analysis

In data processing, Statistical Product and Service Solutions (version 25.0 Inc., Chicago, IL, USA) statistical software was used. Categorical and continuous data were reported in the form of percentage and median with interquartile range (IQR). Mann-Whitney *U* and chi-square (*χ*^2^) tests, as appropriate, were used to assess the differences between two groups at a statistical significance level of 0.05.

## Results

A total of 1858 TSCI cases were included; the median age was 38 (IQR = 27–49) years old; and the median rehabilitation LOS was 117 (IQR = 55–239) days.

### Participant characteristics: fall vs non-fall etiologies

Table 1 summarizes the demographic, injury, and medical characteristics patients with fall-induced TSCI (41.7%, 775/1858) compared with patients with non-fall-induced TSCI (58.3%, 1083/1858). Specifically, motor vehicle collisions represented 31.3%; struck by object, 13.9%; work-related, 5.8%; sports-related, 4.0%; assault, 1.3%; and others, 1.7%. Generally, patients with fall-induced TSCI (40 years old, IQR = 28−51) were older than those with non-fall-induced TSCI (37 years old, IQR = 26−47) (*P* < 0.001).

**Table 1:** Characteristics of participants resulted from fall and non-fall (*N* = 1858)

Characteristics	Non-fall group	Fall group	*P* value
	*n* = 1083 (%)	*n* = 775 (%)	
Age (years)			*χ*^2^ = 29.251, *P* < 0.001
0–14	90 (8.3%)	29 (3.7%)	
15–29	284 (26.2%)	192 (24.8%)	
30–44	367 (33.9%)	238 (30.7%)	
45–59	285 (26.3%)	250 (32.3%)	
60 and above	65 (5.3%)	66 (8.5%)	
Sex			*χ*^2^ = 11.502, *P* = 0.001
Male	819 (75.6%)	637 (82.2%)	
Female	264 (24.4%)	138 (17.8%)	
Neurological level of injury			*χ*^2^ = 2.130, *P* = 0.144
Tetraplegia	460 (42.5%)	303 (39.1%)	
Paraplegia	623 (57.5%)	472 (60.9%)	
Severity of the injury			*χ*^2^ = 2.975, *P* = 0.085
Incomplete	529 (48.8%)	410 (52.9%)	
Complete	554 (51.2%)	365 (47.1%)	
Complication			*χ*^2^ = 0.850, *P* = 0.357
Yes	720 (66.5%)	531 (68.5%)	
No	363 (33.5%)	244 (31.5%)	
Rehabilitation LOS (IQR)	126 (58, 268)	100 (50, 194)	*P* < 0.001*

*IQR* interquartile range, *LOS* length of stay

*Mann-Whitney *U* test

Statistically significant differences were found between patients with fall- and non-fall-induced TSCI with regard to age range, gender, rehabilitation LOS rather than neurological level of injury, severity, and complications (Table 1). Patients with fall-induced TSCI tended to be older, usually male (43.8% male vs 34.3% female).

### Participant characteristics: low fall vs high fall etiologies

Falls from buildings were the most common cause of fall-induced TSCI (22.7%), followed by scaffolding (16.8%), and slipping, tripping, and stumbling at the same height (15.0%), falls on same level due to collision with another person (5.2%), all on and from stairs and steps (4.4%). Other falls from height accounted for 23.1%, but the specific situation was not clear due to the incomplete medical records.

Table 2 summarizes the demographic, injury, and medical characteristics of patients with fall-induced TSCI. Specifically, 212 cases (27.4%) were caused by low falls and 563 cases (72.6%) were by high falls. Patients with low fall-induced TSCI were older than those with high fall-induced TSCI [52 (IQR = 37.25–60) years old vs 37 (IQR = 27.00–46) years old, *P* < 0.001]. Patients were transferred to rehabilitation hospitals 47 (IQR = 24–124) days after injury.

**Table 2:** Characteristics of participants resulted from low-fall and high-fall (*N* = 775)

Characteristics	Low fall group	High fall group	*P* value
	*n* = 212 (%)	*n* = 563 (%)	
Age (years)			χ^2^ = 170.039, *P* < 0.001
0–15	17 (8.0%)	12 (2.1%)	
16–30	20 (9.4%)	172 (30.6%)	
31–45	33 (15.6%)	205 (36.4%)	
46–60	88 (41.5%)	162 (28.8%)	
61 and above	54 (25.5%)	12 (2.1%)	
Sex			*χ*^2^ = 2.332, *P* = 0.127
Male	167 (78.8%)	470 (83.5%)	
Female	45 (21.2%)	93 (16.5%)	
Neurological level of injury			*χ*^2^ = 134.059, *P* < 0.001
Tetraplegia	153 (72.2%)	150 (26.6%)	
Paraplegia	59 (27.8%)	413 (73.4%)	
AIS grade			χ^2^ = 103.288, *P* < 0.001^#^
A	49 (23.1%)	316 (56.1%)	
B	31 (14.6%)	108 (19.2%)	
C	53 (25.0%)	67 (11.9%)	
D	79 (37.3%)	69 (12.3%)	
E	0 (0.0%)	3 (0.5%)	
Cases with spine surgery	186 (87.7%)	555 (98.6%)	*χ*^2^ = 43.168, *P* < 0.001
Vertebrae fracture or dislocation			*χ*^2^ = 275.529, *P* < 0.001
Yes	92 (43.4%)	538 (95.6%)	
No	120 (56.60%)	25 (4.40%)	
Major vertebrae fracture or dislocation			*χ*^2^ = 49.240, *P* < 0.001^#^
C1–C2	3 (3.2%)	1 (0.2%)	
C3–C7	50 (53.8%)	124 (23.0%)	
T1–T4	0 (0.0%)	10 (1.9%)	
T5–T9	11 (11.8%)	52 (9.7%)	
T10–L2	28 (30.1%)	330 (61.3%)	
L3–L5	1 (1.1%)	21 (3.9%)	
Complication			*χ*^2^ = 0.422, *P* = 0.516
Yes	149 (70.3%)	382 (67.9%)	
No	63 (29.7%)	181 (32.1%)	
Other combined injuries			
Extremity bones fracture	11 (5.2%)	131 (23.3%)	*χ*^2^ = 33.638, *P* < 0.001
Craniocerebral injury	38 (17.9%)	129 (22.9%)	*χ*^2^ = 2.267, *P* = 0.132
Thoracic injury	6 (2.8%)	111 (19.7%)	*χ*^2^ = 34.258, *P* < 0.001
Abdominal injury	1 (0.5%)	28 (5.0%)	*χ*^2^ = 8.665, *P* = 0.003
Pelvis fracture	0 (0.0%)	27 (4.8%)	*χ*^2^ = 10.534, *P* = 0.001
≥ 1 extraspinal lesions	50 (23.6%)	300 (53.3%)	*χ*^2^ = 54.857, *P* < 0.001
≥ 2 extraspinal lesions	6 (2.8%)	101 (17.9%)	*χ*^2^ = 29.545, *P* < 0.001
Median time from injury to rehabilitation (IQR)	41 (21, 110.5)	50 (25,126)	*P* = 0.043*
Rehabilitation LOS	78.5 (33, 150.25)	112 (58, 215)	*P* < 0.001*

*AIS* American Spinal Injury Association Impairment Scale, *IQR* interquartile range, *LOS* length of stay.

*Mann-Whitney *U* test

^#^Fisher’s exact test

Patients with low fall-induced TSCI were more likely to suffer from tetraplegia (72.2%) and have incomplete injuries (76.9%). In contrast, patients with high fall-induced TSCI were more likely to suffer from paraplegia (73.4%) and have complete injuries (56.1%).

A total of 630 patients had vertebral fractures and dislocations, accounting for 81.3% of all cases. Patients with high fall-induced TSCI were more likely to have vertebral fractures or dislocations (95.6% vs 43.4%, *P* < 0.001) than those caused by low falls, which were mainly located at the thoracolumbar vertebral level (T10–L2, 61.3%). Among the 145 TSCI cases without vertebral fractures, 82.6% were resulted from a low fall. Overall, 48 cases were diagnosed as SCI without radiographic abnormality, 51 cervical spinal stenosis, and 36 cervical disc herniation or clamping.

During the rehabilitation hospitalization, 531 patients (68.5%) suffered from complications, and no difference between patients with fall- or non-fall-induced TSCI was found. However, patients caused by low falls and high falls were all prone to complications, such as intestinal dysfunction, urinary tract infection, neuropathic pain, and respiratory infection.

Patients with high fall-induced TSCI had a higher risk of combined injuries than patients with low fall-induced TSCI (53.3% vs 23.6%; *χ*^2^ = 54.857, *P* < 0.001), and the same were in extremity bones fracture, thoracic injury, abdominal injury, and pelvis fracture. Craniocerebral injury was common in patients with low fall-induced TSCI, accounting for 17.9% of low-fall cases.

### Different neurological level of injury characteristics: low fall vs high fall etiologies

**Fig. 1:**
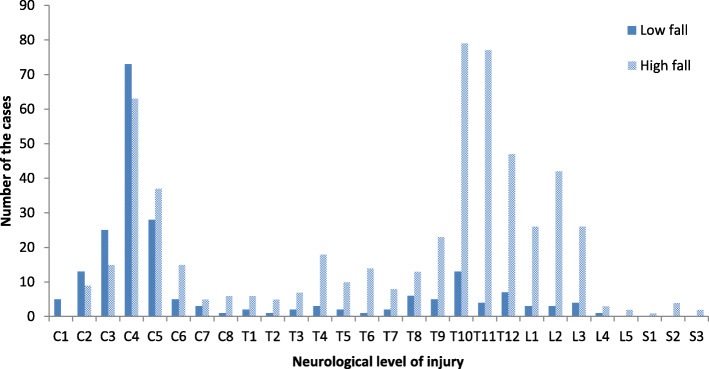
Number of cases with different neurological levels of injury (*n* = 775)

As shown in the Fig. 1, there were two peaks in patients with fall-induced TSCI, including at the cervical (C4–T1, 31.5%) and lower thoracic (T9–T12, 32.9%) spinal cord. Patients with low fall-induced TSCI were more likely to have cervical level-related TSCI, especially at C4 (34.4%). Patients with high fall-induced TSCI were likely to have lower thoracic level-related TSCI (T9–T12, 40.1%); however, injury at the C4 levels was also common (11.2%).

### Occupation characteristics: low fall vs high fall etiologies

A total of 657 patients had specific occupations before injury, accounting for 84.8% of total TSCI cases (173 low fall-induced TSCI and 484 were high fall-induced TSIC). The proportion of manual laborers and subsistence farmers with TSCI caused by a high fall was considerably higher than that caused by a low fall (37.4% vs 17.9% and 22.7% vs 8.7%, respectfully). The total proportion of patients with low fall- and high fall induced TSCI that identified as ‘retired’ represented 22.5% and 1.4%, respectfully, of total cases. Details are shown in the Table 3.

**Table 3:** Proportion of patients with different occupations (*N* = 657)

Occupations	Low fall group	High fall group	Total
	*n* = 173 (%)	*n* = 484 (%)	*N* = 657 (%)
Manual laborers	31 (17.9%)	181 (37.4%)	212 (32.3%)
Subsistence farmers	15 (8.7%)	110 (22.7%)	125 (19.0%)
Unemployed	38 (22.0%)	78 (16.1%)	116 (17.7%)
Retired	39 (22.5%)	7 (1.4%)	46 (7.0%)
Students	7 (4.0%)	34 (7.0%)	41 (6.2%)
Staff	17 (9.8%)	23 (4.8%)	40 (6.1%)
Freelancers	11 (6.4%)	14 (2.9%)	25 (3.8%)
Civil servants	8 (4.6%)	7 (1.4%)	15 (2.3%)
Professional skill workers	4 (2.3%)	9 (1.9%)	13 (2.0%)
Active duty soldiers	0 (0.0%)	11 (2.3%)	11 (1.7%)
Self-employed	2 (1.2%)	8 (1.7%)	10 (1.5%)
Enterprise administrators	1 (0.6%)	2 (0.4%)	3 (0.5%)

## Discussion

Falls is a common cause of SCI. Different demographic characteristics may have different causes of injury, which may also lead to different clinical prognoses. Here, the characteristics of TSCI caused by falls and non-falls were different. Patients with fall-induced TSIC were older and had shorter rehabilitation LOS than those with non-fall-induced TSCI. Compared with patients who caused by low falls, patients who fell from height were younger; more likely to suffer from paraplegia, vertebral fractures or dislocations, and combined injuries and undergo spine surgery; and had longer time from injury to rehabilitation and longer hospitalization LOS.

Despite the rapid economic development in China, falls was still the most important cause of TSCI. Previous studies have shown that among TSCI patients admitted to Tianjin Medical University General Hospital from 1998 through 2009, the main causes of TSCI were falls (low fall, 35.2%; high fall, 16.7%), followed by motor vehicle collision (36.4%) [14]. This survey shows that the main causes were still falls in 2011–2019, followed by motor vehicle collision (31.3%). However, there were far more patients who fell from height than those who fell from low places (low fall, 11.4%; high fall, 30.3%). The infrastructure and housing construction in China are still in the ascendant, but security protection is not standardized due to the large and complex migrant workers market [15]. Ministry of Housing and Urban-Rural Development of China reported 415 high fall accidents in 2019 with an increase of 8.4% over 2018 and accounting for 53.69% of all safety accidents [16]. For a long time in the future, falling from height is likely to remain an important cause of spinal cord injury in the future.

Therefore, it is necessary to clarify the mechanism and characteristics of spinal cord injury. An epidemiological survey on falls from height while clearing snow in Akita prefecture, Japan from 2015 to 2018 found that among patients with spinal fractures, lumbar fractures are most likely to occur, accounting for 73.8% [17]. Yokota M et al. reported that 33.4% of patients fell from height had spinal fractures, and 6.3% of ground-level falls had spinal fractures [18]. Among patients with high fall-induced TSCI, the 16–45 age group accounted for 69.6% of all cases. Overall, 37.4% of patients with high fall-induced TSCI were manual laborers and 22.7% were subsistence farmers. Compared with patients with low fall-induced TSCI, more patients with high fall-induced TSCI suffered from paraplegia (73.4% vs 27.8%) and thoracolumbar spine fractures (T10–L2; 61.3%). Ivancic PC applied a human spine specimen model inside a crash dummy from a high place and observed clinically relevant compression and flexion-distraction injuries and largest injurious compressive load at the lower vertebra as compared with the upper vertebra [19]. The thoracolumbar vertebrae are the transition site of the thoracic vertebrae and lumbar vertebrae. The orientation of the facet joints changes, resulting in increased stress in the vertebrae. The body weight shifts from the front to the back of the vertebrae, making the region more vulnerable when the patient falls from a high place [20].

Falling below 1 m is considered minor trauma; however, in geriatric patients, cervical spine fractures are relatively common [21, 22]. In addition with the presence of comorbidities including osteoporosis, osteopenia, degenerated osseous changes could synergistically impact the severity of injury pattern when low fall occurs [23]. Low fall results in an acceleration-deceleration force or change in velocity that causes significant head and neck movement, which would typically lead to blunt cervical spine injuries [24]. Thus, patients with this relatively lower imparted energy injury tend to suffer from tetraplegia and/or incomplete injuries. A multi-center survey in the USA reported that among the patients with SCI caused by falling on the same level, 87.8% had cervical SCI [11]. Another investigation reported that patients with spinal trauma due to a fall from standing, 43% had cervical spine injuries [25] and falls from standing level represent 47% of C2 fractures in patients over 70 [26].

Low fall plays a major role in patients who are 60 years or older, accounting for 41.2%. Feng et al. also reported that the proportion of the low-fall group was increasing constantly in China [14], and low fall is the majority causes in the patients older than 65. This may be due to osteoporosis, decreased muscle strength and the increased risk of falls in the elderly [27]. However, a report in Chongqing, China, found that among 996 elderly patients with spinal fractures, the most common fracture areas were in the lumbar (48.4%) and thoracic (43.0%) regions, but not the cervical region (8.2%) [28]. This apparent discrepancy in results may be related to the fact that 55.4% of the study participants were not caused by a fall, and that only 7.5% of patients in our cohort were 61 years or older. For the elderly who are admitted to the hospital due to low falls, clinicians need to consider whether there is cervical spine injury or tetraplegia, so as to perform appropriate physical examinations, imaging examinations, and treatments.

The LOS serves as an index to evaluate the costs of patients, which is very important for patients with SCI [29]. In our survey, the time from injury to rehabilitation and rehabilitation LOS patients with high fall-induced SIC were longer than those with low fall-induced TSCI. Patients with complete TSCI had higher risks of complications than patients with incomplete injuries, which may lead to longer LOS [30, 31]. In addition, it has been shown in previous studies that patients with complications after TSCI have longer LOS [32]. While the presence of some complications does affect the patient’s LOS, such as pressure ulcers [33]. High falls are associated with a bigger force to spine and more likely to cause severer injuries, such as complete SCI, leading to longer LOS [34].

With aging of the population in China, increasing numbers of elderly people are living alone, and thus more attention should be paid to prevent fall in community and home environment [35]. Home safety checklist should be generated [36] and older people’s living environments should be improved, such as removing clutter, loose carpets and uneven floor surfaces, and providing good lighting, hand rails, and appropriate toilets and beds [37]. Community-based health efforts, such as awareness, and physiotherapy, occupational therapy, and physician-led interventions, will also have a positive role in preventing of falls [38]. For younger adults, especially for manual laborers and subsistence farmers, safety awareness is very important, including proper equipment use and training, safety inspections, and testing, as well as environmental modifications.

Rehabilitation has become the key health strategy of the twenty-first century [39]. Nevertheless, due to the uneven distribution of medical resources, many patients with SCI cannot continue to receive specialized institution-based rehabilitation after being discharged from the emergency department [40, 41]. Among 3487 patients with SCI in Northwest China, only 15.14% received long-term rehabilitation treatment [9]; which brings great difficulties to the management and prevention of secondary complications of patients with SCI. Attention should also be paid to the prevention of falls in patients with SCI. An investigation of multicenter specialized rehabilitation centers in Sweden showed that 50% of patients reported more than two falls in 1 year and fall prevention programs should be focused on ambulatory, younger, and more active individuals who are at the highest risks for recurrent falls [42]. Early assessment of the risk of falls in patients with SCI can provide targeted training for patients with SCI [43].

### Limitations

As a retrospective study in a single rehabilitation center, the accuracy and completeness of documentation in the medical records was assumed. Second, patients had undergone surgery in other hospitals before transfer to the rehabilitation center, which made it impossible to obtain surgical and follow-up information. Moreover, admission bias also exists for individuals in this cohort as all were rehabilitation inpatients and different studies had inconsistent standards for high fall and low fall, which limited epidemiological characteristic comparisons with other surveys. Finally, more detailed data are needed to support the in-depth study of the included variables.

## Conclusion

Falling is one of the leading causes of TSCI and high fall is becoming more common. Patients with fall-induced TSCI were older and a longer rehabilitation LOS than those with non-fall-induced TSCI. Among the patients with TSCI caused by falls, those who fell from height were younger and more likely to suffer from paraplegia, combined injuries, sever injuries, and had longer hospitalization LOS and time from injury to rehabilitation, compared with patients who fell from a low height or at the same level. Attention not only should be paid to high falls for the severe injury and longer hospitalization, but also low falls due to the higher neurological level of the injury and the aging of population in China.

## Data Availability

The datasets generated and/or analyzed during the current study are not publicly available due to personal privacy but are available from the corresponding author on reasonable request.

## Abbreviations

AIS: American Spinal Injury Association Impairment Scale
IQR: Interquartile range
LOS: Length of stay
SCI: Spinal cord injury
TSCI: Traumatic spinal cord injury
χ^2^: Chi-square
